# Supplementary material for: Does a mother’s childbirth experience influence her perceptions of her baby’s behaviour? A qualitative interview study
Source: PLoS One. 2023 Apr 6;18(4):e0284183. doi: 10.1371/journal.pone.0284183 (PMC10079033; doi:10.1371/journal.pone.0284183)
Supplement: Attachment — Submitted filename: FIN review PLOS_22_24651 oct 2022.docx [file pone.0284183.s001.docx]

**Does a mother’s childbirth experience influence her perceptions of her baby’s behaviour? A qualitative interview study**

Reviewers comments

This is an interesting paper, addressing an important area. It is well written, with good logical progression, and is generally comprehensive. However, I do have some comments and questions about some aspects of the text. Fundamentally, the paper seems to be trying to address too many issues at once. I would suggest that the team carefully consider the primary focus, and trim the paper down so that that focus is very clear and consistent.

I have made a number of comments about the small sample size. This is not a problem if there are sound and saturated themes, and the claims are being made about the nature of these themes. However, in this case, a number of claims of causation/association are made in the data, and these do need larger sample sizes and more representative samples in order to be substantiated.

*Fit between the aims and the methodological approach*

The text seems to suggest that there is already evidence of an association between birth experience and women’s perceptions of infant behaviour:

*Indeed, our recent quantitative study of approximately a thousand mothers [30] found that the mother’s subjective birth experience and her postnatal mood were associated, and that together they predicted self reported infant behavioural style, commonly known as temperament [31, 32]*

Given this existing information, what this study seems to add is a description of what women believe about the nature of their experiences and perceptions of infant temperament. I would be inclined to take out suggestions of empirical cause and effect, as that is not really the epistemological focus of qualitative research. However, exploring whether mothers **believe** that the two are related, as in the second part of the text below, is appropriate: so I suggest that this is the emphasis throughout, as in the proposed amendments to the aim below:

*Consequently, the aim of the current study was to further explore ~~how~~ mothers’ subjective childbirth experiences ~~might influence~~ and their perceptions and interpretations of their baby’s behaviour, and whether they believed that the two were related.*

This framing encompasses the results reporting both what the women say they believe, and the ‘researchers view’ in which you provide a critique of what they say they believe in the light of how they talk about their perceptions. It may be useful to structure the paper a bit more clearly at these two different levels of analysis (and I would suggest that some of the more quantitative and ‘causative’ aspects of the paper could be deleted to allow for a more in-depth exploration of the nuances between what is described by the participants, and how they interpret what they describe).

*Reflexivity*

Reflexivity is an important corner stone of qualitative research, as it enables the reader to understand which lens(es) are applied to the design, data collection, analysis and interpretation of the study, from the perspective of the researcher pre-suppositions before the study started. A paragraph or two describing the standpoints and beliefs of the research team members in relation to the topic of the paper would add considerable to the credibility and rigour of the study.

For instance, the assumption in the abstract and in some parts of the text is that childbirth interventions are associated with traumatic birth. While this is true for some women, there is good evidence that the more important factor overall is how women are treated during labour and birth. Reflecting on this may change both the framing of the issue and the solutions proposed.

*Methodology*

This is generally well explained, and the approach to thematic analysis is appropriate. However, I wonder why the data were analysed by birth perceptions as a group and then by perceptions of infant behaviour as a group, rather than looking ‘vertically’ across each birth and infant perception story by individual, to illustrate the nuances of the different patterns of experience and perception?

As above, I am not sure about the relevance of the quantification of the data where this occurs.

Did the team look for disconfirming data during the analysis?

P 8 The statement is made that the analysis applies *Lincoln and Guba’s concept of ‘thick description’, with transferability applying to similar contexts.*

The key reference for thick description should really be Geertz: for example, *Geertz, C. (1973). Thick Description: Towards an Interpretative theory of culture. The Interpretation of Cultures, 3-31.*

This refers to more than just describing the data, and includes a wider analysis of the underlying relations, interactions, semiotics and etc of the context in which the individuals in the research are situated. I’m not sure this was done in the current study?

P8 Do the experience categories of ‘positive’ mean that only positive things were said, negative only negative, and mixed everything else? This is a rather reductionist approach in qualitative terms.

I’m also not sure if the coding of positive or mixed or negative birth or easy mixed or difficult infant behaviour was done on the basis of one final question about the overall birth experience, or on the researchers interpretation of the whole set of responses from each woman:

*A ‘positive’ experience, while grounded in the most recent WHO Intrapartum Guidelines [34], is defined here according to subjective maternal experience. ‘Positive’ experiences were mostly spontaneous physiological births happening in a calm, supportive environment. ‘Mixed’ births were considered a mixture of positive and negative experiences, such as feeling unsupported or experiencing separation during or post birth. ‘Negative’ experiences generally involved obstetric complications or postnatal difficulties. Maternal perceptions of childbirth as a positive, mixed or negative experience were confirmed with mothers during interviews by asking them how they rated their birth experience overall.*

*Sample*

The demographics seem to cover a good range. Are they representative of the population? What about ethnicity? This is covered in the limitations but it might be worth making a note about the sample representativeness at the outset of the results, to remind the reader.

*Data collection*

The questions and example prompts given in Table two are appropriate.

How long did the interviews last (the range would be useful here)?.

The following statement is a good indication of the trustworthiness of the analysis. How was it ascertained that no new or meaningful concepts were being generated?:

*Data collection stopped when it was judged that no new and meaningful concepts were being ‘generated’ from the data [40].*

*Results*

The results make good use of quote material.

Table 3: Part one doesn’t look like themes that have emerged from the data – they are demographics. How many had each of these experiences? (to interpret the credibility of the claims made later). Part two is a bit more nuanced: but what about Joy? Delight? Did these not feature?

Given the current debates in the UK about terminology for types of labour and birth, there may be a risk in dichotomising assumptions about ‘natural’ and ‘clinical’ birth, and of making assumptions about place and type of birth on very small samples. Indeed the key point seems to more about the fit or gap between expectations and experience than about place or type of birth? Women’s experiences related to different types and places of birth are already well described in various studies – the contribution to knowledge here seems to be more about whether women say that the way they perceived their labour and birth overall has influenced the behaviour of their infants. Indeed, there does seem to be some disconfirming data for the claims made about type of birth (p17) in the account of a ‘natural’ birth that was then associated with distress (although this seemed to be more about the (lack of) postnatal care than the mode of birth): *Mother 10, natural hospital birth, baby in Neonatal Intensive Care Unit [NICU] post birth)*

Table 5 speaks to the topic but the ‘n’ is needed, and ‘birth mode’ or ‘pain relief’ are demographics not themes – ‘Baby mirrors mother’ or ‘maternal caregiving’ are closer to a thematic concepts.

N of how many? Words such as ‘often’ are used throughout, or statements such as ‘waterbirths were…’. Does this mean the majority of respondents said these things? How many waterbirths were there? I would suggest that throughout the text should be more cautious. For example:

p23 *‘…..often enabled their baby to be calm and settled*’ is better as ‘*some who reported overall positive births linked this experience with their babies being calmer and more settled’*

And the opposite with the following quote about forceps and the behaviour of the baby:

*Conversely, birth complications sometimes led to pain, distress and unsettled baby behaviours.*

I would suggest this is changed to

*‘some women suggested that specific birth events (such as forceps delivery) might be linked to specific baby behaviours’:*

In both cases, in terms of disconfirming data, were there any accounts from women with a birth coded as ‘positive’ or ‘negative’ who said anything different to the statements above?

P24 I’m not sure why this quote is used with reference to labour pain/pain relief:

*She’s definitely a more clingy baby than my first. She does not like to be put down, she doesn’t lie down, she’s quite sicky... She won’t settle away from me.”*

It maybe that a longer section of this quote shows that the mother is associating this behaviour with labour pain/use of pain relief, and, if so, the quote needs to be expanded to show this.

P25 It is good to see a counterfactual case here – but is the mother associating the baby’s colic with her birth experience? Or is she just saying that she (the mother) found it a bit harder to cope than she might given her birth experience?

*“I think I found her quite difficult because I was a little bit shell-shocked after the birth… in the early days… She was really hard to console for no reason other than maybe feeling a bit colicky…”* (Mother 8, MLU waterbirth, no medication)

P27 As noted above, I would suggest that, at least based on the data from this study, mode of birth/interventions are not part of the psychological experience measure, but that this should be built on the mothers perceptions. The sample size in this study is not big enough to be certain that mode of birth/interventions are definitively associated with the outcome, in the absence of other contextual aspects.

P29 The following comment is interesting, but the ‘n’ is very small for a statement of association, and the demographics are not necessarily representative. Any statement like this needs to be made very cautiously, and with the imperative that the findings need to be verified with a much larger sample:

*Notably, no mother who had a spontaneous physiological birth described her baby’s behaviour as difficult, with the majority describing an easy baby. Conversely, almost half of all clinical births were associated with a description of a difficult baby, with less than a quarter describing their baby as easy.*

Table 9 also interesting, but, again, it should be noted that this is a small and skewed sample, and the assumptions need to be checked with larger samples. It isn’t quite clear what the direction of effect is. Does a difficult baby reify the birth experience as negative, or vice versa?

Based on prior studies, the statement below is important, but, based on an n of two, it cannot be transferable from this study. It would be better to frame this as ‘*Two women had planned interventions (one CS and one induction) and both reported positive perceptions’*

*‘…The remaining 25% of mothers with positive perceptions of their birth (n = 2) had experienced a planned intervention, such as a planned induction or CS’*

**Discussion**

The discussion could be shorter. There are also some new findings here. Some of the assumptions seem to be quite far from the actual data, or, at least, don’t seem to take account of possible alternative explanations. This is less likely to happen when the team have previously considered their reflexivity positions, and deliberately searched for disconfirming data.

For example, in relation to the two comments below: what about the counter-factual? Might the fact that the newborn is perceived to be unsettled be linked to something else other than the birth? Difficulties in feeding, lack of postnatal support, maternal worry about financial security, for instance: meaning that there is no cognitive dissonance in their accounts in relation to infant behaviour and the birth?:

P30

*After outlining a physically traumatic birth, some mothers proceeded to describe their newborn as unsettled, without attributing their baby's behaviour in any way to the birth. This might be due to maternal guilt, disassociation or denial after a difficult birth experience [52], and therefore, may not have wished to perceive or disclose a potential association between the birth and their baby’s behaviour*

P31

*Consequently, the lack of consistency in maternal responses could be caused by cognitive dissonance stemming from a desire to believe that their baby had experienced a positive start to life regardless of the type of birth they had experienced*

P32 Yes, the statement below is correct, the numbers are too small to make any claims – I would suggest deleting this comment, or reframing it as I have suggested

*Although the sample numbers are too small to draw any definitive conclusions, the n = waterbirths were ~~generally~~ associated with maternal perceptions of calmer infants, while the n= unsettled forceps birth infants were considered to have residual pain and distress*

P32 The concept of Potential Triggers is interesting in the discussion

P33 The findings underpinning the statements made below are only represented by one or two quotes (or no direct quotes). The highlighted phrases in the first para indicate where strong claims are made that should be rendered much more nuanced and tentative (and there are similar observations for the other paras selected):

*Birth environment was considered important in relation to maternal and infant wellbeing. Noisy, brightly lit environments increased maternal stress whereas births taking place at home or in a MLU were generally reported as more physically comfortable and psychologically positive experiences. Women who experienced an in-labour transfer from home or MLU to hospital could find it difficult to acclimatise to their new environment, particularly if the transfer involved interventions which raised their stress levels. Unexpected or traumatic circumstances affected maternal reports of infant behaviour. Therefore, the birth environment seemed to have profound psychological as well as physical impacts on the mother and infant.*

*Midwifery services were often stretched despite the most recent National Institute for Health and Care Excellence (NICE) recommendations of one-to-one care for all labouring women [51]. Inconsistent care and noticeable staff shortages on the postnatal ward were experienced as distressing, with staff shortages on wards known to negatively impact on quality of care [63, 64]. The resulting maternal distress could adversely impact newborn infant behaviour. In contrast, women who had a calm, supportive birth were more likely to attribute their baby’s calm, settled behaviour to the birth environment.*

Do the data actually say this?:

P35 *c) Health professional authority Perceived ‘health professional authority’ occurred when women experienced a loss of control. Participants who were not fully informed or felt ‘coerced’ into consenting to unplanned interventions often had regrets around their birth.*

There are other statements in the discussion that don’t seem to relate to the findings above. I suggest you check for and amend or remove these throughout. I would suggest as above that the focus of the discussion stays on maternal perceptions of their births and the behaviour of their infants.

P40 The limitations section is rather long. Also, in qualitative terms, and given the aim of this paper to understand women’s reported experiences, I don’t think the following is a limitation, since the whole point (as I understand it?) is to capture women’s subjective experiences: *However, parents may also be influenced by prenatal expectations, and their emotional involvement naturally precludes them being entirely objective observers of their infant’s temperament*

The limitations about the sample are accurate, but are not always reflected in the discussion of the meaning and potential transferability of the study findings.

P39 suggest changing:

*Whilst this study deepens our understanding of how physical and psychological birth experiences may affect early infant temperament…*

To:

*Whilst this study deepens understanding of* ***womens perceptions*** *of how their physical and psychological birth experiences may affect the temperament of their infant…*

*Conclusion*

*P40 –* While it may be true that what the researcher sees as ‘difficult’ is not necessarily in line with the woman’s perception and that women MAY be engaging in self-deception for self-protection, this is an assumption that values the researcher interpretation over what the woman herself says. In fact, the woman may feel no need for self-protection – she may simply telling it as it is from her perspective. Both points of view need to be discussed here.

It is important to explain and reference in the discussion how better one-one care and emotional support during childbirth help with women’s perceptions of their birth, their infants behaviour, and any associations between these two, if this is to be a key conclusion of the study.
